# Decoding ferroptosis in ischemic stroke: key genes and the therapeutic potential of acupuncture

**DOI:** 10.3389/fnagi.2025.1506276

**Published:** 2025-06-09

**Authors:** Chunxiao Wu, Zhirui Xu, Qizhang Wang, Shuping Zhu, Chunzhi Tang

**Affiliations:** ^1^Shenzhen Hospital of Integrated Traditional Chinese and Western Medicine, Shenzhen, China; ^2^Shenzhen Clinical College of Integrated Chinese and Western Medicine, Guangzhou University of Chinese Medicine, Shenzhen, Guangdong, China; ^3^Clinical Medical of Acupuncture, Moxibustion and Rehabilitation, Guangzhou University of Chinese Medicine, Guangzhou, Guangdong, China

**Keywords:** ferroptosis, ischemic stroke, acupuncture, bioinformatics analysis, differentially expressed genes (DEGs)

## Abstract

**Background:**

Ferroptosis has been reported to be associated with the development of ischemic stroke; however, comprehensive investigations of ferroptosis-associated genes are lacking. Herein, key differentially expressed genes (DEGs) related to ferroptosis in ischemic stroke were identified and validated and the potential mechanism of acupuncture in treating ischemic stroke was explored through bioinformatics analysis and experiments.

**Methods:**

High-throughput RNA sequencing was performed to identify DEGs between cerebral ischemic injury tissue and normal brain tissue in mice. Subsequently, differentially expressed ferroptosis-related genes (DE-FRGs) were identified by intersecting the DEGs with a ferroptosis database. Functional enrichment analysis was conducted to investigate potential signaling pathways involved in ischemic stroke, while protein-protein interaction (PPI) analysis was used to explore interactions among the DE-FRGs. Hub genes were identified using the random forest algorithm, and their RNA expression levels were validated via RT-qPCR in sham-operated, MCAO model, and acupuncture groups.

**Results:**

The results showed that one hundred twenty-seven DE-FRGs were identified by comparing cerebral ischemic injury tissue with normal brain tissue in mice. KEGG enrichment analysis revealed that these DE-FRGs were primarily involved in the ferroptosis pathway, autophagy–animal pathway, apoptosis pathway, HIF-1 signaling pathway, and longevity-regulating pathway. The top 10 hub DE-FRGs selected through the PPI analysis and random forest algorithm included SLC3A2, FTH1, MAP1LC3A, SLC40A1, TFRC, TMSB4X NRAS CD82 CD44, and PTPN18. Nine genes were confirmed to be significantly differentially expressed between the sham and MCAO mouse models. Moreover, FTH1, SLC40A1, NRAS, CD82, and PTPN18 levels significantly increased after acupuncture in ischemic stroke mice.

**Conclusion:**

The ferroptosis pathway, autophagy–animal pathway, apoptosis pathway, HIF-1 signaling pathway, and longevity-regulating pathway were identified as crucial pathways associated with ferroptosis in cerebral ischemic stroke. Bioinformatics analysis and RT-qPCR suggested that FTH1, SLC40A1, NRAS, CD82, and PTPN18 might serve as potential key targets underlying the antiferroptotic effects of acupuncture on ischemic stroke.

## Introduction

Ischemic stroke is the second leading cause of death and the most common cerebrovascular disease worldwide, with an increasing incidence and a high disability rate, seriously affecting patients’ quality of life and increasing the burden on families and society according to the latest Global Burden of Disease Study (GBD 2019 Stroke Collaborators, 2021). Ischemic occlusion reduces blood flow to brain regions, resulting in brain edema, neuroinflammation, neural cell death, loss of neuronal function, and neurological deficits (Xiong et al., 2018). Recent studies have focused primarily on elucidating the mechanisms underlying ischemic stroke pathophysiology. These mechanisms include cellular excitotoxicity, loss of homeostasis, free radical-mediated toxicity, cytotoxicity, mitochondrial dysfunction, oxidative stress, neuroinflammation, BBB impairment, complement activation of glial cells and cell death processes. Cell death is a vital pathophysiology throughout cerebral ischemia, and among these cell death processes, ferroptosis, a programed cell death-like process, has been confirmed to be attributable to cell death resulting from ischemic stroke (Gelderblom et al., 2009; Macrez et al., 2011; Lai et al., 2014; Datta et al., 2020; Jia et al., 2021; Qin et al., 2022; Bersano and Gatti, 2023). Notably, previous findings indicated that excessive iron accumulation triggered ferroptosis while contributing to increased mitochondrial damage in neuronal cells and infarct volume (Park et al., 2015). In addition, other studies have demonstrated that knocking out the GPX4 gene induces ferroptosis and exacerbates cerebral ischemic stroke (Zhang et al., 2018). Furthermore, other investigations suggest that dysfunction within system xc^–^ occurs during ischemic stroke, leading to glutamate toxicity and inhibiting GPX4 production, ultimately triggering ferroptosis (Massie et al., 2015; Hsieh et al., 2017). However, many ferroptosis-associated genes have not yet been investigated. Therefore, further studies are required to explore potential ferroptosis-related genes involved in ischemic stroke to identify potential therapeutic targets for its treatment (Bu et al., 2021; Zhang et al., 2021).

Acupuncture has demonstrated promising clinical efficacy in managing ischemic stroke. Previous studies have reported that acupuncture can enhance motor and sensory functions and mitigate cell ferroptosis and cerebral infarction size, thereby ameliorating neurological deficits associated with ischemic stroke (Wu C. et al., 2021; Wang et al., 2023). However, the specific regulatory mechanism of acupuncture has not been fully elucidated, particularly regarding its modulation of ferroptosis-related target genes in the context of ischemic stroke.

Therefore, in this study, we aimed to investigate differentially expressed genes (DEGs) in cerebral ischemic injury tissue and normal brain tissue from mice using high-throughput RNA sequencing (RNA-seq). Subsequently, the identified DEGs were intersected with a ferroptosis dataset to identify ferroptosis-related DEGs, and key DE-FRGs were screened by the random forest algorithm. Additionally, functional annotation and protein-protein interaction (PPI) analyses were conducted to elucidate potential mechanisms underlying cerebral ischemic stroke. Finally, to test our hypothesis and explore the role of acupuncture in treating ischemic stroke by modulating ferroptosis mechanisms, key ferroptosis-related DEGs were selected to validate their RNA expression levels in a mouse MCAO model and an intervention (acupuncture) model using RT-qPCR. Our findings will contribute to a better understanding of the involvement of ferroptosis in ischemic stroke pathogenesis and will provide a preliminary understanding of the relationship between ferroptosis mechanisms and acupuncture in the treatment of ischemic stroke.

## Materials and methods

All the animal procedures met the ethical requirements for experimental animal welfare, in accordance with the National Institute of Animal Protection guidelines, and were approved by the Ethics Committee of Guangzhou University of Chinese Medicine. This study was conducted in accordance with the ARRIVE guidelines.^1^

### Animals

Adult male C57BL/6 mice (aged 8-10 weeks, SPF grade) were purchased from the Animal Experimental Center of Guangzhou University of Chinese Medicine. First, the mice were randomly divided into two groups for RNA sequencing: MCAO model group and sham group (each group included three mice). After obtaining the RNA sequencing results, the animals were randomly assigned to the following three groups (sham group, MCAO model group, and MCAO model + EA group) to test the hypothesis and explore the relationship between ferroptosis mechanisms and acupuncture in the treatment of ischemic stroke. All the mice were kept under controlled temperature and humidity conditions with a 12-h light–dark cycle, and food and water were available *ad libitum*.

### MCAO model

The cerebral ischemia/reperfusion injury (MCAO) model was established based on previously described methods (Bertrand et al., 2017). In brief, first, the mice were anesthetized intraperitoneally with isoflurane to minimize pain; then, the left common carotid artery, left internal carotid artery, external carotid artery and vagus nerve were exposed through a midline incision under an ordinary microscope, and the corresponding separation and ligation were performed. A suture plug with a length of approximately 11 ± 0.5 mm was inserted through the internal carotid artery to block the blood supply to the middle cerebral artery. One hour after ischemic infarction, the suture plug was slowly removed to restore the blood supply and achieve ischemia-reperfusion injury. Mice in the sham operation group were subjected to the same surgical method as described above, but middle cerebral artery embolization was not performed.

### Acupuncture methods

“Baihui, Dazhui” and “Zusanli, Quchi” were selected as the intervention acupoints. The choice of these acupoints as an intervention strategy is primarily informed by pertinent research findings, which indicate that these specific acupoints can enhance motor function deficits and mitigate pathological brain damage associated with ischemic stroke. Additionally, acupuncture at these points has been shown to modulate cellular ferroptosis in the context of ischemic stroke (Wu C. et al., 2021; Pu et al., 2024; Zhu et al., 2024). The acupoint positioning was based on the animal acupoint selection standards in “Experimental Acupuncture and Moxibustion.” After routine disinfection with iodine and alcohol, the operator inserted a disposable sterile Φ0.16 × 13 mm acupuncture needle into the acupoint and then connected the electroacupuncture instrument. “Baihui” and “Dazhui” were connected to electrical stimulation together as one group, and “Zusanli” and “Quchi” were connected as another group. The electrical wave was continuous, the current was 1 mA, the stimulation frequency was 2 Hz, and the tissue around the point was measured by slight shaking. Each mouse underwent acupuncture intervention for 7 consecutive days for 20 min each session.

### RNA-seq

Briefly, total RNA was extracted from the brain tissue of each group using TRIzol (Invitrogen, Carlsbad, CA, United States) according to the manufacturer’s protocol. Subsequently, RNA quality control, including RNA concentration and integrity, was performed using a NanoDrop spectrophotometer and an Agilent 2100 Bioanalyzer (Thermo Fisher Scientific, MA, United States). mRNA was purified using oligo (dT)-linked magnetic beads and transformed into cDNA, and then, libraries were constructed based on the standards of the RNA sequencing library manufacturer. Finally, the PCR products were heated, denatured, and circularized with a splint oligo sequence to obtain the final library. The final library was further amplified to create DNA nanoballs (DNBs) with more than 300 copies. DNBs were then loaded into a patterned nanoarray, and single-ended 50-base reads were generated on the BGISeq500 platform (BGI-Shenzhen, China).

### Differential expression analysis

The raw data obtained by sequencing were filtered using SOAPnuke (v1.5.2) to filter out reads containing adapters, reads with an unknown base N content greater than 10%, and low-quality reads, and finally, clean data were obtained (Chen et al., 2018). The clean data were aligned to the reference genome using HISAT2 (v2.0.4) software and aligned to the reference gene set using Bowtie2 (v2.2.5) (Langmead and Salzberg, 2012; Kim et al., 2015). Then, the expression levels of the genes were calculated by RSEM (v1.2.8) (Li and Dewey, 2011). Differentially expressed genes (DEGs) were detected by using DESeq2 (v1.4.5) and the following criteria: *Q*-value ≤ 0.05 or FDR ≤ 0.001 (Love et al., 2014). We also obtained a dataset of 259 ferroptosis-related genes from a ferroptosis database (FerrDb),^2^ and these genes were cross-referenced with the above DEGs to identify DE-FRGs. Venn diagrams and heatmaps of DEGs associated with ferroptosis were generated using the R software packages “VennDiagram,” “heatmap,” and “ggplot2.”

### Gene set enrichment analysis of ischemic stroke

Gene set enrichment analysis (GSEA) was conducted to explore the signaling pathways related to ischemic stroke, and GSEA of the DEGs was performed using the “clusterProfiler,” “msigdbr,” and “ggplot2” packages. The significance thresholds for demonstrating statistically significant GSEA results were set at a *p*-value < 0.05 and a *q*-value (FDR) < 0.25. The top 10 results are shown in the GSEA plots.

### Investigation of ferroptosis-related genes by GO and KEGG pathway enrichment analyses

Functional enrichment analysis of the DE-FRGs was performed using the Database for Annotation, Visualization and Integrated Discovery (DAVID). In addition, GO enrichment and KEGG pathway enrichment analyses of the DE-FRGs were performed to determine the potential functions of the genes using the “stringi,” “ggplot2,” and “dplyr” packages. The top 20 results were displayed in enrichment plots.

### PPI analysis and correlation analysis of ferroptosis-related DEGs

The STRING online database^3^ was utilized to construct a protein–protein interaction (PPI) network of DE-FRGs. The genes are represented with nodes, and the interactions between the genes are shown as edges using Cytoscape software. MCODE was used for gene network cluster analysis to identify the key network modules. Spearman correlation analysis was performed using the “Corrplot” package to detect correlations between the differentially expressed ferroptosis-related genes (ferroptosis pathway genes and non-ferroptosis pathway genes). A *p*-value less than 0.05 was considered to indicate a statistically significant correlation. The hub genes in the ferroptosis pathway and non-ferroptosis pathway were further screened by a random forest algorithm.

### Random forest sequencing

The expression levels of the hub genes in the ferroptosis pathway and non-ferroptosis pathway were calculated via a random forest algorithm. Hub genes were screened through the R package “random forest.” To determine the optimal error rate and the optimal number of stable trees as optimal parameters, the error rate of each tree from 1 to 500 trees was calculated. Then, random forests were used to screen key genes, and the minimum Gini (MDG) in the random forest algorithm was applied to quantify the classification accuracy and dimensional significance values. The genes with the 5 highest MDG scores were regarded as hub genes and were used for subsequent experimental verification.

### qRT-PCR analysis

After 7 days of acupuncture intervention (sham: *n* = 3; MCAO model: *n* = 3; MCAO + EA: *n* = 3), the brain tissue of each mouse was extracted for qRT-PCR to analyze the hub genes. In brief, the total RNA from each sample was collected using TRIzol, and the RNA concentration and purity were detected using a NanoDrop 2000 spectrophotometer. Total RNA was then reverse transcribed into cDNA template via reverse transcription. Real-time fluorescence PCR (RT-QPCR) was performed using SYBR Green qPCR Master Mix according to the instructions of the kit and conducted on a Bio-Rad C1000 Touch™ Thermal Cycler. The mRNA expression of the hub genes was calculated using the 2-ΔΔCt method. GAPDH was used as a housekeeping gene, and each experiment was repeated three times. The primer sequences are shown in Supplementary Table 1.

### Statistical analysis

The data were analyzed using SPSS 25.0, and all the data are presented as means ± standard errors. If the data were normally distributed and had homogeneous variances, one-way analysis of variance (ANOVA) was used for comparisons among multiple groups (sham group, model group, and model + EA group), and Bonferroni correction was used for *post hoc* pairwise comparisons. If the assumption of homogeneity of variance was not met, Dunnett’s T3 test was used for *post hoc* pairwise comparisons; if the data did not follow a normal distribution, the data are presented as M (P25, P75), and the non-parametric Kruskal-Wallis H rank sum test was used for comparisons among multiple groups. A *p*-value less than 0.05 was considered to indicate statistical significance.

## Results

### Gene expression analysis

**FIGURE 1:**
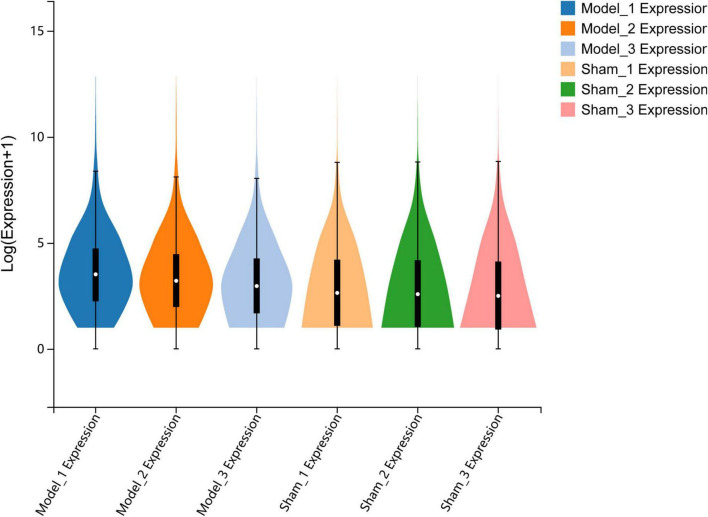
Quantitative analysis of gene expression. The violin plot shows the overall range and distribution of FPKM values of gene expression in all included samples.

Total RNA was extracted from the sham group and MCAO control group, and RNA sequencing was subsequently performed using the DNBSEQ platform. The total number of clean reads from each sample in the sham and MCAO groups ranged from 43.95 to 45.5 million, and the total mapping genome ratio ranged from 95.25 to 96.07%. The gene expression of each sample was represented by fragments per kilobase of transcript sequence per million base pairs sequenced (FPKM). The FPKM of each sample was represented with a Violin plot, which suggested that the distribution of gene expression was approximately consistent with that of each sample, demonstrating that the gene expression data of each sample were reliable and reproducible (Figure 1).

### Differential expression of ferroptosis-related DEGs

**FIGURE 2:**
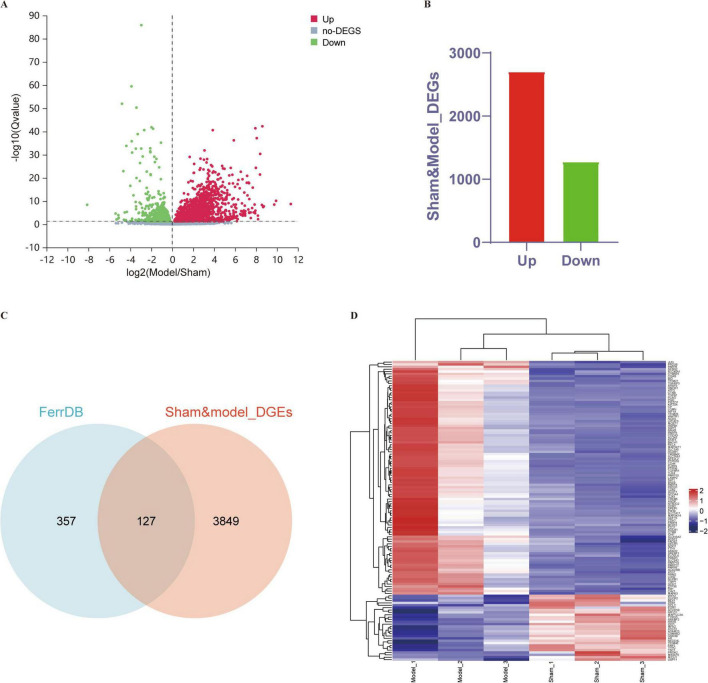
DE-FRGs between the sham and MCAO model groups. **(A,B)** Volcano plot and histogram displaying significantly differentially expressed genes in ischemic stroke patients. Red dots represent the upregulated genes, and green dots denote the downregulated genes, with thresholds of *Q*-value ≤ 0.05. **(C)** Venn diagram displaying DE-FRGs. We compared the ferroptosis dataset with the RNA-seq data of MCAO mice to identify DE-FRGs. **(D)** Heatmap displaying the expression of the 127 DE-FRGs in ischemic stroke patients. Red bricks represent the more highly expressed DE-FRGs, and purple bricks indicate lower expression.

A total of 3,976 differentially expressed genes (DEGs), i.e., 2,702 upregulated genes and 1,274 downregulated genes, were identified between the sham and MCAO control groups. A volcano plot and Venn diagram of the DEGs are shown in Figures 2a,b. We intersected 256 ferroptosis-related genes classified as ferroptosis drivers, ferroptosis suppressors, and ferroptosis markers from the ferroptosis online database with DEGs to identify ferroptosis-related DEGs, and ultimately, 127 DEGs were detected as DE-FRGs, and the specific expression of 127 DE-FRGs was visualized in a heatmap (Figures 2C,D). This visualization suggests that these differential genes may serve as potential key regulators of ferroptosis in the context of ischemic stroke. The specific information of DE-FRGs is shown in Supplementary Table 2.

### Gene set enrichment analysis

**FIGURE 3:**
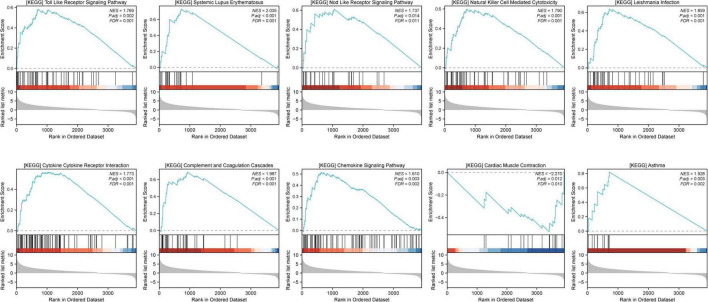
Gene set enrichment analysis (GSEA) was performed to explore the signaling pathways related to ischemic stroke. GSEA results were considered significant if the *p*-value was < 0.05 and the q value (FDR) was < 0.25.

GSEA was performed to compare distinct pathways between the sham and MCAO model groups, and the top 10 GSEA pathways were as follows: cytokine receptor interaction, systemic lupus erythematosus, complement and coagulation cascades, Leishmania infection, natural killer cell-mediated cytotoxicity, Toll-like receptor signaling pathway, asthma, chemokine signaling pathway, cardiac muscle contraction, and Nod-like receptor signaling pathway (Figure 3), indicating that these pathways may be closely related to ischemic stroke. The remaining GSEA plots are displayed in Supplementary Figure 1.

### Functional enrichment analysis of DE-FRGs

**FIGURE 4:**
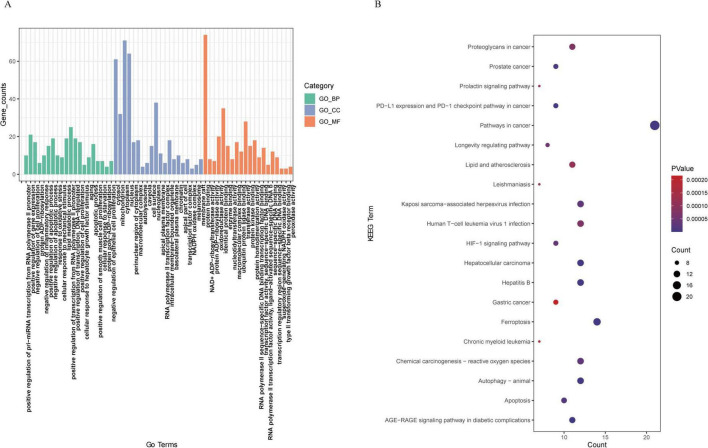
Functional annotation of DE-FRGs. **(A)** Gene Ontology (GO) enrichment analysis of the enriched DE-FRGs. **(B)** KEGG pathway enrichment analysis of the enriched DE-FRGs.

GO and KEGG pathway analyses were subsequently conducted to explore the potential biological functions and pathways of the DE-FRGs between the sham group and MCAO model group. The results showed that the biological processes of GO enrichment were significantly enriched, such as positive regulation of pri-miRNA transcription from the RNA polymerase II promoter, regulation of gene expression, regulation of cell proliferation, protein ADP-ribosylation, regulation of inflammatory response, response to oxidative stress and regulation of apoptotic process. The top enriched cellular components were cytosol, mitochondrion, cytoplasm, nucleus, and perinuclear region of cytoplasm. The molecular function of GO revealed that the DE-FRGs were enriched in protein binding, NAD + ADP-ribosyl transferase activity, protein ADP-ribosylase activity, oxidoreductase activity and identical protein binding. In addition, KEGG enrichment analysis of the DE-FRGs revealed that ferroptosis-related DEGs were mainly involved in the ferroptosis pathway, the AGE-RAGE signaling pathway in diabetic complications, autophagy–animal, apoptosis, chemical carcinogenesis–reactive oxygen species, the HIF-1 signaling pathway and the longevity-regulating pathway. The top 20 genes were displayed in both GO and KEGG pathway enrichment plots (Figure 4).

### Construction of the protein–protein interaction network and visualization of ferroptosis-related DEGs

**FIGURE 5:**
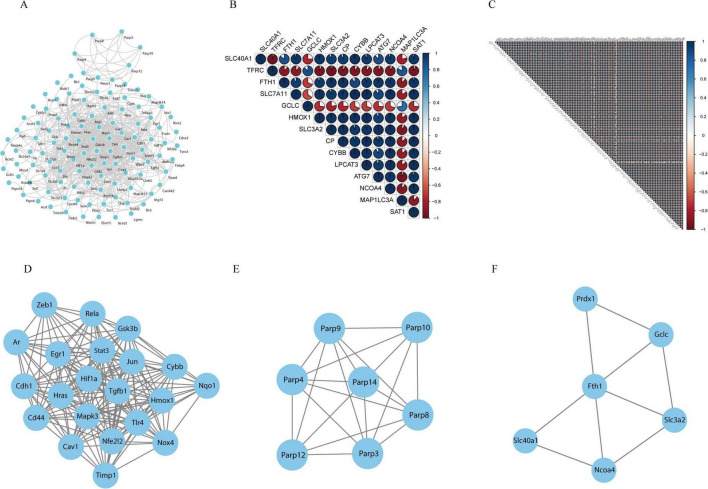
Protein-protein interaction (PPI) analysis of the 127 differentially expressed ferroptosis-related genes. **(A)** PPIs among the differentially expressed ferroptosis-related genes. **(B)** Spearman correlation analysis of the differentially expressed ferroptosis-related genes in the ferroptosis pathway. **(C)** Spearman correlation analysis of the differentially expressed ferroptosis-related genes in the non-ferroptosis pathway. **(D–F)** The three key modules were identified by MCODE, which was used to identify network gene clustering.

To explore the interactions among all DE-FRGs, a protein–protein interaction (PPI) network was constructed and visualized using Cytoscape. Figure 5A shows the interaction networks of the DE-FRGs, which included 111 nodes and 592 edges. Spearman correlation analysis revealed strong interactions between the differentially expressed ferroptosis-related genes in both the ferroptosis pathway and the non-ferroptosis pathway (Figures 5B,C). In addition, the PPI networks were divided into different clusters of gene networks using the application program MCODE in Cytoscape. The first main cluster, with a score of 17.3, consisted of 21 genes; the second cluster, with a score of 6.667, contained 7 genes, and the third cluster, with a score of 3.6, was composed of 6 genes, including FTH1, NCOA4, PRDX1, GCLC, SLC40A1, and SLC3A2, which were mainly linked to the ferroptosis pathway (the specific genes are shown in Figures 5D-F).

### Identifying the key DE-FRGs using a random forest classifier

**FIGURE 6:**
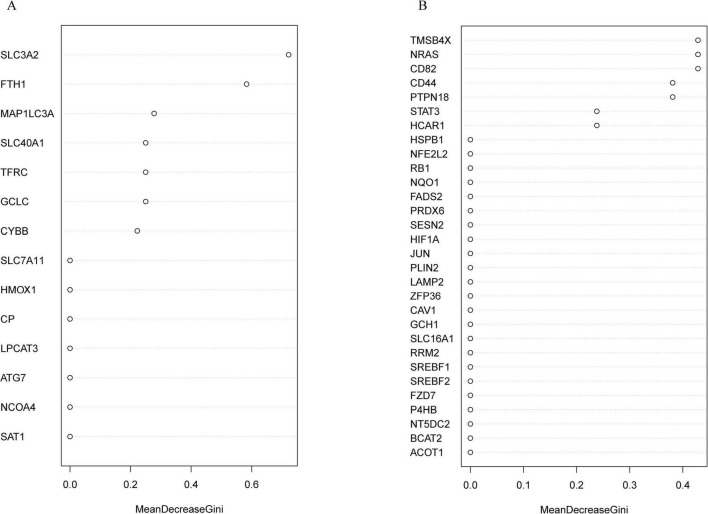
Identification of the most important key DE-FRGs by using a random forest classifier. **(A)** The top-ranking DE-FRGs, as determined by MeanDecreaseGini values, in the ferroptosis pathway. **(B)** The top-ranking DE-FRGs, as screened by MeanDecreaseGini values, in the non-ferroptosis pathway.

The randomForest package can be used to predict and identify the importance of a variable. The most important key DE-FRGs were identified using the MeanDecreaseGini indicator. The top five ranked DE-FRGs in the classical ferroptosis pathway according to the MeanDecreaseGini value included the SLC3A2, FTH1, MAP1LC3A, SLC40A1, and TFRC genes (Figure 6A). The top five DE-FRGs in the non-ferroptosis pathway are listed in Figure 6B and include TMSB4X, NRAS, CD82, CD44, and PTPN18). This suggests that these candidate genes may serve as crucial core markers for the identification of ischemic stroke. The top 5 genes in the ferroptosis and non-ferroptosis pathways were further validated by RT-qPCR.

### Validation of hub gene expression by qRT-PCR

**FIGURE 7:**
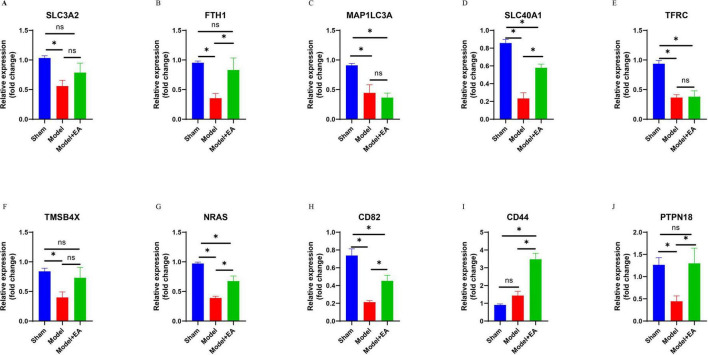
Validation of 10 important key DE-FRGs via RT-qPCR. **(A–J)** Illustrate the expression levels of SLC3A2, FTH1, MAP1LC3A, SLC40A1, TFRC, TMSB4X, NRAS, CD82, CD44, and PTPN18 across the respective groups. The statistical graph shows the fold change in the mRNA expression of the DE-FRGs in each group, normalized to the GAPDH level. The data are presented as means ± SEMs; *n* = 3 mice/group (with three technical replicates). *p < 0.05, *^ns^p* > 0.05.

The top five genes related to the ferroptosis pathway according to the random forest plot were assessed by qRT-PCR. The results showed that the expression levels of SLC3A2, FTH1, MAP1LC3A, SLC40A1, and TFRC were lower in the MCAO model group than in the sham group (*P* < 0.05). Compared with those in the MCAO group, the expression of these five genes tended to differ between the acupuncture intervention group and the MCAO group; however, the expression of only FTH1 and SLC40A1 in the acupuncture group significantly increased compared with that in the MCAO control group (*p* < 0.05). In addition, the expression levels of TMSB4X, NRAS, CD82, and PTPN18 in the non-ferroptosis pathway were lower than those in the sham group, and the expression levels of NRAS, CD82, CD44, and PTPN18 in the non-ferroptosis pathway were higher in the MCAO control group after acupuncture intervention (*p* < 0.05) (Figure 7). The results demonstrated that the expression levels of FTH1, SLC40A1, NRAS, CD82, CD44, and PTPN18 were upregulated in mice with ischemic stroke following acupuncture treatment.

## Discussion

In this study, we identified key ferroptosis-related genes involved in ischemic stroke, explored the potential mechanism of ferroptosis in cerebral ischemic stroke and preliminarily investigated the relationship between ferroptosis and acupuncture in the treatment of ischemic stroke. In the present study, 127 DE-FRGs were identified from the overlap of genes identified via RNA-seq (MCAO model versus sham group) and in FerrDb datasets, indicating that these DE-FRGs might be key targets of pathological changes caused by ischemic stroke.

KEGG enrichment analysis was subsequently conducted to investigate the differentially expressed genes related to ferroptosis, revealing their involvement in key pathways, including ferroptosis, autophagy, apoptosis, and the hif-1 signaling pathway. Ferroptosis is an iron-dependent type of regulated necrosis characterized by extensive lipid peroxidation-mediated membrane damage; it primarily encompasses two major pathways: a transporter-dependent pathway (e.g., TFRC, SLC40A1, SLC7A11, NCOA4, and FTH1) and an enzyme-regulated pathway (GPX4, ACSL4, and ALOXs), which are regulated by iron metabolism, lipid metabolism and oxidation and antioxidant systems (Tang et al., 2021; Zhang et al., 2023). Previous studies have demonstrated substantial iron accumulation in damaged nerve cells, along with ferroptosis in an ischemic stroke model (DeGregorio-Rocasolano et al., 2018; Liu et al., 2020a; Zhang et al., 2021). Inhibition of the ferroptosis signaling pathway through targeted interventions or the use of specific inhibitors can effectively reduce iron deposition and enhance lipid peroxidation while suppressing nerve cell ferroptosis in ischemic stroke. This approach holds promise for limiting infarct size and ameliorating neurological deficits associated with ischemic stroke (Tuo et al., 2017; Guan et al., 2019). Increasing evidence suggests that targeting genes involved in the ferroptosis signaling pathway may serve as effective therapeutic strategies for the treatment of ischemic stroke (Ratan, 2020; Liu Y. et al., 2022). Our study highlighted the importance of autophagy as a crucial pathway linked to ferroptotic processes in ischemic stroke pathogenesis. Previous research has revealed intricate interactions between autophagy and ferroptosis, specifically illustrating how iron accumulation and lipid-mediated ROS trigger autophagy, which subsequently amplifies either iron overload or lipid peroxidation under certain conditions, leading to the eventual induction of ferroptotic cell death (Lee et al., 2023). Previous studies have also demonstrated the involvement of selective autophagy in the regulation of brain iron accumulation and lipid peroxidation following ischemic processes, thereby promoting ferroptotic cell death (Liu et al., 2020a; Liu et al., 2020b; Zhou et al., 2020). Therapeutic strategies targeting the interplay among autophagy, iron, and ROS in ferroptosis could offer promising new directions for treating ischemic stroke (Liu et al., 2020a; Sun et al., 2021). The results of our study indicated that the HIF-1 signaling pathway is associated with ferroptosis and plays a crucial role in ischemic stroke. Related research has shown an intrinsic link between HIF-1 signaling and the occurrence of ferroptosis in ischemic stroke (Liu C. et al., 2022). The HIF-1 pathway might induce cellular iron overload through TfR1 regulation, leading to cellular ferroptosis (Guo et al., 2023; Sanguigno et al., 2023). Conversely, other studies have demonstrated that the activation of the Hif-1 signaling pathway may reduce lipid peroxidation and inflammatory responses while exerting neuroprotective effects against cerebral ischemia. These divergent findings might be attributed to disparate regulatory mechanisms governing the response to ischemic stroke mediated by HIF-1α (Cui et al., 2021; Dong et al., 2022). Further investigation is required to elucidate the specific regulatory role of HIF-1α and ferroptosis in ischemic stroke.

Our study revealed significant differences in the expression of the SLC3A2, FTH1, MAP1LC3A, SLC40A1, and TFRC genes between the MCAO model and sham model groups. These genes are predominantly associated with the ferroptosis signaling pathway and collectively contribute to the regulation of cellular iron homeostasis. Among these genes, SLC40A1 (FPN1) plays a crucial role in iron metabolism by regulating the efflux of iron ions and maintaining cellular iron homeostasis. Previous studies have shown that the expression of SLC40A1 is significantly reduced in MCAO models, which aligns with our findings (Wang P. et al., 2021 Luo et al., 2023). The downregulation of SLC40A1 expression inhibits iron efflux, leading to intracellular iron overload and subsequent ferroptosis. Increasing the expression of SLC40A1 through targeted interventions can potentially reduce nerve cell ferroptosis and improve neurological deficits (Wang P. et al., 2021; Luo et al., 2023). Our study also demonstrated that acupuncture treatment, compared with the control, upregulated the expression of SLC40A1. This finding suggested that acupuncture may regulate ischemic stroke by targeting the iron metabolism-related gene SLC40A1. Previous studies have demonstrated that acupuncture can regulate the expression of iron metabolism-related genes (upregulated SLC40A1) in ischemic stroke. This regulation has been shown to reduce iron overload, inhibit ferroptosis, and reverse cerebral ischemic injury, effects that are consistent with our findings (Liang et al., 2021; Wang et al., 2023). TFRC is another iron metabolism-related gene that is involved mainly in iron influx. However, recent studies have indicated that TFRC might exert dual effects on iron metabolism, namely, TFRC could cause iron overload and might also promote iron clearance when iron overaccumulation occurs in the brain after ischemic stroke (Zhang and Pardridge, 2001; Fang et al., 2022). The specific regulatory mechanism involved in ischemic stroke requires further investigation. In addition, our results showed that FTH1 was significantly decreased in cerebral ischemic injury tissue. Moreover, acupuncture intervention tended to increase the expression of FTH1. FTH1 acts as an iron storage protein that helps reduce iron deposition and oxidative stress reactions while decreasing ferroptosis. When cerebral ischemic injury occurs, the abnormal expression of the cargo transporter NCOA4 can lead to the selective degradation of FTH1 and the release of a large amount of iron ions to enter the intracellular iron pool, thereby aggravating cellular ferroptosis (Quiles Del Rey and Mancias, 2019). The findings of related studies were also in line with our results, which demonstrated that acupuncture could increase the expression of FTH1 mRNA and thereby inhibit ferroptosis (Li et al., 2021). SLC3A2 serves as one of the core transporter subunits comprising system Xc^–^, which facilitates extracellular cystine exchange while simultaneously binding to intracellular glutamate. Through a series of catalytic chemical reactions within cells, γ-glutamylcysteine synthetase (γ-GCS) is formed from these interactions, ultimately leading to glutathione (GSH) formation for protection against ferroptosis in ischemic stroke (Dixon et al., 2012; Fujii et al., 2020; Fang et al., 2022). The findings of our study indicate that the downregulation of SLC3A2 expression increases the occurrence of ferroptosis in cerebral ischemic injury, which is consistent with the results of previous research (Bu et al., 2021; Zhang et al., 2021; Fang et al., 2022; Guo et al., 2023). However, further investigations are needed to elucidate the specific mechanism by which SLC3A2 regulates ferroptosis in ischemic stroke.

Our study revealed that the NRAS, CD82, and PTPN18 genes were significantly differentially expressed between the sham group and the model group and between the model group and the acupuncture group. Although these genes are not classic members of the ferroptosis pathway, they directly or indirectly interact with genes involved in this pathway. NRAS is a member of the RAS family and has been implicated in regulating iron metabolism and reducing erastin sensitivity and ferroptosis through the modulation of TFRC and iron storage protein expression (Miyake et al., 2020; Wu S. et al., 2021). Enhancing the expression of the NRAS gene could be an important target for acupuncture in the treatment of ischemic stroke, as it may inhibit ferroptosis by decreasing iron accumulation and reducing erastin. Previous bioinformatic analysis using SVM-RFE algorithms suggested a role for CD82 in modulating ferroptosis (Hu et al., 2023). CD82 exerts a positive effect on SLC40A1 and GPX4 expression, thus modulating ferroptosis (Liu et al., 2021). However, related studies on CD82-mediated ferroptosis regulation in ischemic stroke are lacking, and CD82 might be a novel target of acupuncture-mediated ferroptosis regulation in ischemic stroke. Increased PTPN18 levels have been shown to activate the GPX4/xCT signaling pathway and protect against ferroptotic cell death (Wang H. et al., 2021). Therefore, acupuncture may enhance the expression of PTPN18, thereby exerting inhibitory effects on ferroptosis in ischemic stroke. However, due to the intricate molecular mechanisms underlying electroacupuncture (EA) in ischemic stroke, further *in vivo* experiments are warranted for additional validation. Furthermore, there is a necessity for additional research involving larger sample sizes to improve reproducibility and validate our findings.

## Conclusion

In summary, the ferroptosis pathway, autophagy–animal pathway, apoptosis pathway, HIF-1 signaling pathway, and longevity-regulating pathway were identified as crucial factors closely associated with ferroptosis in the context of cerebral ischemic stroke. Furthermore, through bioinformatics analysis and RT-qPCR, we successfully identified nine ferroptosis-related genes involved in the development of ischemic stroke. Among them, FTH1, SLC40A1, NRAS, CD82, and PTPN18 emerged as potential key targets underlying the antiferroptotic effects of acupuncture on ischemic stroke. However, further experimental validation of the mechanism by which acupuncture regulates ferroptosis in ischemic stroke is still needed.

## Data Availability

The datasets presented in this study can be found in online repositories. The names of the repository/repositories and accession number(s) can be found in the article/Supplementary material.

## Footnotes

1
https://www.nc3rs.org.uk/arrive-guidelines

2
zhongnan.org

3
https://string-db.org/
