# Supplementary material for: Decoding ferroptosis in ischemic stroke: key genes and the therapeutic potential of acupuncture
Source: Front Aging Neurosci. 2025 Jun 9;17:1506276. doi: 10.3389/fnagi.2025.1506276 (PMC12183209; doi:10.3389/fnagi.2025.1506276)
Supplement: Supplementary file 1 [file Table_1.docx]

**Supplementary file Online Content**

[**Supplementary Table S1: Detailed primer sequences of genes 2**](#_Toc163553700)

[**Supplementary Table S2: Detailed information of the Ferroptosis differentially expressed genes of ischemic stroke 3**](#_Toc163553701)

[**Supplementary figure1: Gene set enrichment analysis (GSEA) was performed to explore the related signaling pathways in ischemic stroke. 6**](#_Toc163553702)

# Supplementary Table S1: Detailed primer sequences of genes

Table1a. Detailed primer sequences of genes

| Gene | Sequences |
| --- | --- |
| SLC3A2 | FORWARD: TTCCTCACCTGACCTCTTCTCCTAC  REVERSE: CTCGGAAGTTGAGCACCACCAG |
| FTH1 | FORWARD: TGCCATCAACCGCCAGATCAAC  REVERSE: AGTTCTTCAGAGCCACATCATCTCG |
| MAP1LC3A | FORWARD: GCCTTCTTCCTGCTGGTCAACC  REVERSE: ATCCGTCTTCATCCTTCTCCTGTTC |
| SLC40A1 | FORWARD: TTGGTGACTGGGTGGATAAGAATGC  REVERSE: CAGGATGATTCCGCAGAGGATGAC |
| TFRC | FORWARD: TCGTGGAGACTACTTCCGTGCTAC  REVERSE: TCTTGGAGATACATAGGGCGACAGG |
| TMSB4X | FORWARD: TGACAAACCCGATATGGCTGAGATC  REVERSE:  ACGATTCGCCAGCTTGCTTCTC |
| NRAS | FORWARD: GTGGTTGGAGCAGGTGGTGTTG  REVERSE: AGTATGTCCAGCAGGCAGGTCTC |
| CD82 | FORWARD: TCATCGCACAGGTGACCGTAGG  REVERSE: TGGTGGCATTGGCAGTGTAGTTG |
| CD44 | FORWARD: CTCAAGTGCGAACCAGGACAGTG  REVERSE: ATCAGAGCCAGTGCCAGGAGAG |
| PTPN18 | FORWARD: GACGGAGGAAGTGTGAACGCTAC  REVERSE: CCTGGAGGGTCCTGAGAGTGATG |
| GAPDH | FORWARD: CCTCGTCCCGTAGACAAAATG  REVERSE: TGAGGTCAATGAAGGGGTCGT |

# Supplementary Table S2: Detailed information of the Ferroptosis differentially expressed genes of ischemic stroke

| Gene Symbol | log2 (Model/Sham) | Qvalue (Model/Sham) |
| --- | --- | --- |
| CYBB | 6.545437715 | 2.22E-23 |
| CD44 | 3.85453835 | 5.51E-23 |
| CAV1 | 1.540479388 | 8.14E-19 |
| PTPN6 | 3.369994848 | 3.98E-16 |
| PARP9 | 2.661067187 | 9.93E-16 |
| ATF3 | 4.722253638 | 7.73E-15 |
| PARP14 | 2.98306273 | 3.97E-14 |
| IDO1 | -3.70184141 | 3.17E-13 |
| LCN2 | 5.118119668 | 3.53E-12 |
| PLIN2 | 4.784656268 | 5.69E-12 |
| HMOX1 | 3.090182228 | 1.06E-10 |
| PARP12 | 1.786168693 | 2.01E-10 |
| NFE2L2 | 1.877583082 | 2.09E-09 |
| TLR4 | 2.904890521 | 2.39E-09 |
| PTPN18 | 2.802677765 | 1.60E-08 |
| JUN | 0.819874876 | 1.97E-08 |
| TGFB1 | 2.915251569 | 3.89E-08 |
| PARP3 | 2.167588375 | 9.96E-08 |
| CTSB | 2.457864446 | 1.63E-07 |
| LGMN | 2.209280067 | 5.31E-07 |
| PARP10 | 2.363923988 | 7.38E-07 |
| PIEZO1 | 2.122961226 | 1.11E-06 |
| TGFBR1 | 1.609682117 | 3.09E-06 |
| CP | 2.088215973 | 3.22E-06 |
| SAT1 | 1.652562149 | 5.74E-06 |
| SLC16A1 | 1.11008039 | 5.75E-06 |
| TIMP1 | 4.920206976 | 8.03E-06 |
| LAMP2 | 1.535678756 | 1.34E-05 |
| TRIM21 | 1.858367807 | 1.37E-05 |
| ZFP36 | 2.399736314 | 1.80E-05 |
| TMSB4X | 1.123632542 | 2.46E-05 |
| CDCA3 | 2.698016948 | 2.60E-05 |
| RRM2 | 2.114226093 | 2.61E-05 |
| CYB5R1 | 0.805073293 | 3.32E-05 |
| PRDX6 | 0.898835442 | 5.86E-05 |
| SLC7A11 | 1.870750243 | 8.68E-05 |
| SLC3A2 | 0.612934856 | 9.91E-05 |
| KIF20A | 2.008144818 | 0.000219 |
| SREBF2 | -0.789064625 | 0.000289 |
| TYRO3 | -0.960972859 | 0.000324 |
| SNX5 | 0.983256519 | 0.000416 |
| TNFAIP3 | 1.180101663 | 0.000481 |
| FABP4 | 5.20265064 | 0.000529 |
| FURIN | 0.756697417 | 0.000789 |
| SREBF1 | 0.819228352 | 0.000867 |
| BEX1 | -0.569417061 | 0.000912 |
| CGAS | 1.658442634 | 0.001005723 |
| BACH1 | 0.956324679 | 0.001046416 |
| MAPK3 | 0.526320711 | 0.001097524 |
| STAT3 | 0.905372027 | 0.001198904 |
| HRAS | -0.735224259 | 0.001359141 |
| MGST1 | 1.605923532 | 0.001429785 |
| ANO6 | 0.99553518 | 0.001820774 |
| SOCS1 | 2.300555578 | 0.001872527 |
| RB1 | 0.458438941 | 0.002180677 |
| NUPR1 | 1.380197046 | 0.0028037 |
| PRDX1 | 0.82818979 | 0.002874691 |
| TFRC | -0.704566403 | 0.002917214 |
| HSPB1 | 1.573403937 | 0.00296856 |
| DDR2 | 1.690781804 | 0.003276213 |
| SLC1A5 | 1.453561342 | 0.003370558 |
| GJA1 | 0.839752822 | 0.003734398 |
| NT5DC2 | 1.423581403 | 0.003836352 |
| ALOX5 | 1.661651783 | 0.004136261 |
| PPARD | 0.560483805 | 0.004863879 |
| GSK3B | -0.49109568 | 0.004895521 |
| PGD | 1.149767303 | 0.004895521 |
| CD82 | 1.176053188 | 0.004952151 |
| RELA | 0.872166796 | 0.005623461 |
| WWTR1 | 0.937602673 | 0.005630353 |
| DPEP1 | 4.638625583 | 0.005839277 |
| CAMKK2 | -1.420107809 | 0.006028297 |
| FZD7 | 1.606991112 | 0.006363944 |
| TSC1 | -0.383887473 | 0.006503413 |
| MAP1LC3A | -0.473671707 | 0.006994652 |
| P4HB | 1.11779708 | 0.007528645 |
| ACADSB | 0.360742843 | 0.007916557 |
| MS4A15 | -5.320880302 | 0.008144479 |
| PARP4 | 0.923425518 | 0.008180002 |
| NRAS | 0.556438457 | 0.008605904 |
| LPCAT3 | 0.985056302 | 0.009013539 |
| PROK2 | -2.29665745 | 0.009216193 |
| NQO1 | 1.017279829 | 0.010388037 |
| ADAM23 | -0.768829397 | 0.01048128 |
| FAR1 | -0.389541764 | 0.010820485 |
| BID | 0.636172282 | 0.011454443 |
| ALDH3A2 | 0.286039473 | 0.011560017 |
| GDF15 | 3.273232547 | 0.011809901 |
| CDH1 | 2.016575899 | 0.011881971 |
| MAP3K14 | 1.103355153 | 0.012275433 |
| ACOT1 | 0.894207902 | 0.013456771 |
| TRIM26 | 0.66178268 | 0.013997255 |
| EGR1 | -1.321470221 | 0.014218777 |
| ELOVL5 | 0.469216235 | 0.015688529 |
| GCH1 | 1.739458205 | 0.01571679 |
| AR | -0.487249421 | 0.015954539 |
| DECR1 | 0.598847562 | 0.016053857 |
| MICU1 | -0.333101926 | 0.016735633 |
| SIRT2 | 0.787131028 | 0.018465394 |
| FTH1 | 0.59270502 | 0.019845972 |
| NCOA4 | 0.420473116 | 0.020256316 |
| FADS2 | 0.334866718 | 0.020907167 |
| HCAR1 | 1.73953395 | 0.021479872 |
| USP11 | -0.312851877 | 0.021647187 |
| ASAH2 | 0.556656081 | 0.022433434 |
| NR1D1 | -0.847907654 | 0.024663395 |
| ACSF2 | 0.603042897 | 0.025860493 |
| NEDD4L | -0.893606557 | 0.02772323 |
| SESN2 | 0.485788359 | 0.02773548 |
| DUOX2 | -3.144858481 | 0.028064783 |
| SLC40A1 | 0.773003953 | 0.028491431 |
| ACVR1B | -0.654430516 | 0.028686952 |
| ATG13 | -0.354381452 | 0.031086739 |
| CREB5 | 0.918487286 | 0.031479618 |
| HIF1A | 0.488792899 | 0.031509583 |
| ATG7 | 0.333915984 | 0.031812896 |
| IDH1 | 0.680740739 | 0.032020579 |
| GCLC | -0.241065768 | 0.032720356 |
| NOX4 | 1.67442335 | 0.033104656 |
| MFN2 | -0.35863466 | 0.035979255 |
| SRSF9 | 0.61253963 | 0.042339917 |
| TRIM46 | -0.732613399 | 0.042438199 |
| ZEB1 | -0.272241358 | 0.042696506 |
| MAP3K11 | 0.646155822 | 0.042851048 |
| BCAT2 | 0.843430217 | 0.044104391 |
| WIPI2 | -0.217023019 | 0.044435857 |
| PARP8 | 0.389941893 | 0.048690001 |

# Supplementary figure1: Gene set enrichment analysis (GSEA) was performed to explore the related signaling pathways in ischemic stroke.


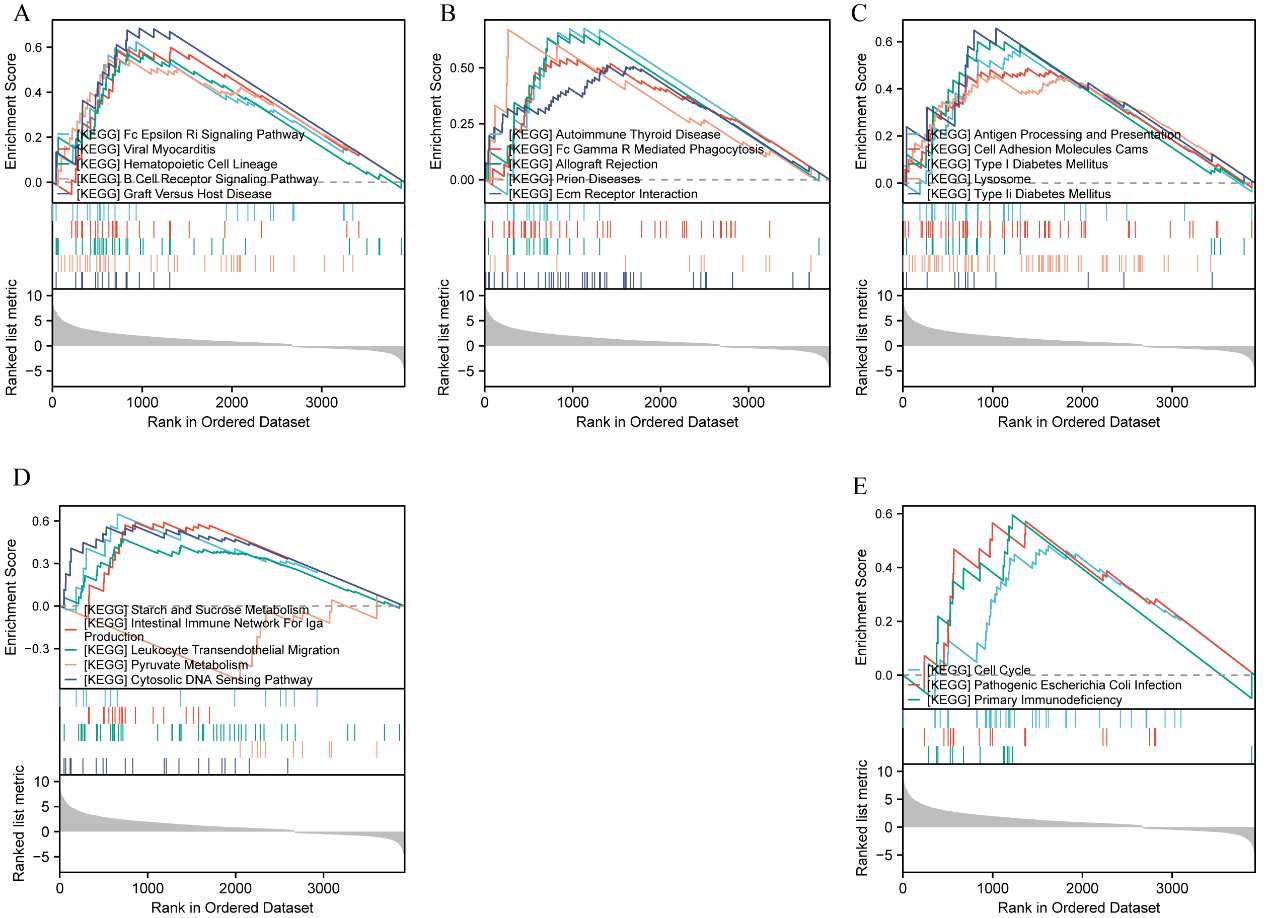


Supplementary figure 1. The remaining GSEA plots was performed to explore the related signaling pathways in ischemic stroke. The significant GSEA was set at p-value<0.05 and q-value (FDR) <0.25.
